# Ethical issues experienced by nurses during COVID-19 in university hospitals^*^

**DOI:** 10.1590/1980-220X-REEUSP-2023-0117en

**Published:** 2023-10-13

**Authors:** Janaína Cassana Mello Yasin, Edison Luiz Devos Barlem, Rosemary Silva da Silveira, Évilin Diniz Gutierres Ruivo, André Andrade Longaray, Laura Cavalcanti Farias Brehmer

**Affiliations:** 1 Universidade Federal do Rio Grande, Rio Grande, RS, Brazil.; 2 Universidade Federal de Santa Catarina, Florianópolis, SC, Brazil.

**Keywords:** Nursing, Coronavirus, Ethics, Nursing, Adult Health, Nursing, Team, Enfermería, Coronavirus, Ética en Enfermería, Salud del Adulto, Grupo de Enfermería, Enfermagem, Coronavírus, Ética em Enfermagem, Saúde do Adulto, Equipe de Enfermagem

## Abstract

**Objective:**

To identify the ethical issues experienced by nurses in the care for patients with COVID-19 and the factors that influence their occurrence.

**Method:**

This is a cross-sectional, quantitative study, carried out between February and May 2022 with 101 nurses from two university hospitals, through the socio-occupational Ethical issues Experienced by Nurses in Emergency Questionnaire, adapted and validated for Brazilians. Descriptive statistical analysis, Pearson’s correlation test and linear regression were performed, adopting p-value.

**Results:**

Ethical issues related to concern and stress in caring for infected patients were evidenced, being influenced by perception of social stigmatization (p = .003) and perception of hospital measures (p = .000). Agreement with infection control measures (4.46) and perception of hospital measures against COVID-19 (3.26) were factors with the highest mean between the constructs.

**Conclusion:**

Nurses are faced with ethical issues in the face of concern and stress in caring for patients with COVID-19, who are affected by social issues and assistance. It is essential to support them, promoting their mental and social well-being to deal with new emergency situations.

## RESUMO

**Objetivo:**

Identificar os problemas éticos vivenciados por enfermeiros no atendimento ao paciente com COVID-19 e os fatores que influenciam a sua ocorrência.

**Método:**

Estudo transversal, quantitativo, realizado entre fevereiro e maio de 2022 com 101 enfermeiros de dois hospitais universitários, através do questionário semiestruturado sociolaboral Ethical Problems Experienced by Nurses in Emergency Questionnaire, adaptado e validado para brasileiros. Realizaram-se análise estatística descritiva, teste de correlação de Pearson e regressão linear, adotando-se p-valor.

**Resultados:**

Evidenciaram-se problemas éticos referente à preocupação e ao estresse no cuidado a pacientes infectados, sendo influenciados pela percepção da estigmatização social (p = ,003) e percepção de medidas hospitalares (p = ,000). Concordância com as medidas de controle de infecção (4,46) e percepção das medidas hospitalares contra a COVID-19 (3,26) foram fatores com maior média entre os construtos.

**Conclusão:**

Os enfermeiros deparam-se com problemas éticos diante da preocupação e do estresse no atendimento aos pacientes com COVID-19, que são afetados por questões sociais e assistências. É essencial apoiá-los, promovendo o seu bem-estar mental e social para lidar com novas situações emergenciais.

## RESUMEN

**Objetivo:**

Identificar los problemas éticos vividos por los enfermeros en el cuidado de pacientes con COVID-19 y los factores que influyen en su ocurrencia.

**Método:**

Estudio transversal, cuantitativo, realizado entre febrero y mayo de 2022 con 101 enfermeros de dos hospitales universitarios, utilizando el cuestionario sociolaboral semiestructurado Ethical Problems Experienced by Nurses in Emergency Questionnaire, adaptado y validado para brasileños. Se realizó análisis estadístico descriptivo, prueba de correlación de Pearson y regresión lineal, adoptando p-valor.

**Resultados:**

Se evidenciaron problemas éticos relacionados con la preocupación y el estrés en el cuidado de pacientes infectados, siendo influenciados por la percepción de estigmatización social (p = .003) y percepción de medidas hospitalarias (p = .000). La concordancia con las medidas de control de infecciones (4,46) y la percepción de las medidas hospitalarias frente a la COVID-19 (3,26) fueron los factores con mayor promedio entre los constructos.

**Conclusión:**

Los enfermeros se enfrentan a problemas éticos ante la preocupación y el estrés en el cuidado de los pacientes con COVID-19, que se ven afectados por cuestiones sociales y asistenciales. Es fundamental apoyarles, promoviendo su bienestar mental y social para afrontar nuevas situaciones de emergencia.

## INTRODUCTION

COVID-19, caused by the 2019 coronavirus (SARS-CoV-2), was declared a pandemic by the World Health Organization (WHO) on March 11, 2020 due to its rapid worldwide spread of exponential growth and since then, have been affecting more than 762 million people, causing more than 6 million deaths worldwide. As of April 4, 2023, Brazil has reported over 37 million confirmed cases of COVID-19 and over 700.000 deaths^(1)^.

The spread of COVID-19 has become a challenging event for everyone, especially for health professionals, who have ­developed their activities on the front lines of fighting the virus, maintaining direct contact with patients infected by the virus and exposed to high viral load, being more vulnerable to the risk of infection^(2)^.

In this context, it was identified during the early stage of COVID-19 in the United States, in which the prevalence of SARS-CoV-2 infection among healthcare workers was 7.3% and, in particular, infections were more common among the nurses^(3)^. In the Netherlands, in just 10 days after the first reported case of COVID-19 in the country, 96 (5%) of 1,796 health workers screened in three hospitals tested positive for SARS-CoV-2^(4)^.

Thus, the scenario of COVID-19 generated a series of ­ethical issues in health professionals’ daily life, especially with nurses, who carry out their activities 24 hours a day directly with suspected or infected patients, being exposed to the risk of infection and transmission to family, colleagues and friends, in addition to knowledge in constant changes about virus behavior and lack of specific treatment to combat the disease, lack of material and human resources, resulting in contamination and infection cross between professionals and patients^(5)^.

In this perspective, research related to previous outbreaks and epidemics showed that, in crisis situations, in addition to the high risk of infection, health professionals experienced ethical issues in the face of the conflict between duty and care, causing stress and generalized fear, resulting in ethical issues^(6,7,8,9)^.

Ethical issues arise when there is a breach of order, i.e., when professionals need to make decisions in the face of ­divergent situations that require caution, consideration and creativity for resolution^(10)^.

It is possible to state that, in the face of emergency situations in public health, nurses experience feelings of fear, uncertainty and stigmatization, which can act as barriers to the performance of their activities in a qualified, empathetic and humane way^(11,12)^. In this context, recognizing the ethical dimension of these ­problems can make professionals less vulnerable to developing mental health problems^(13)^, since nurses are often faced with ethical issues and prefer to remain silent or inert, precisely because they do not know how to behave.

In the international scenario, it is possible to verify that there are studies that explored the risks and ethical issues experienced by nurses during the care of suspected and confirmed COVID-19 patients^(12–16)^. However, in Brazil, research that analyzes the ethical issues experienced by nurses on the front lines of COVID-19 is incipient, being restricted to only ­reflective and literature review studies^(5,17,18)^.

Based on this, the research was justified by the fact that it is fundamental to recognize the ethical issues experienced by nurses during COVID-19 as well as the factors that influence the occurrence of these problems as a way to guide the development and implementation of assessment, support, treatment that consider not only the physical, moral and mental health of professionals in the current context, but also in the experience of new situations of serious infections around the world^(19)^. Given the above, the objective was to identify the ethical issues experienced by nurses in the care of patients with COVID-19 and the factors that influence their occurrence.

## METHOD

### Study Design

This was a cross-sectional study, with a quantitative, descriptive and exploratory approach, developed with nurses working in university hospitals in southern Brazil, based on STrengthening the Reporting of OBservational studies in Epidemiology (STROBE)^(20)^ guidelines and Checklist for Reporting Results of Internet ESurveys (CHERRIES) safety recommendations as guidelines for online data collection^(21)^.

### Site

Data were collected online at two medium-sized federal public university teaching hospitals (H1 and H2), located in two municipalities in southern Brazil. H1 has 237 beds, and H2, 175 beds. The two institutions have Emergency Care Unit, clinical, surgical, intensive care and specific units for suspected or confirmed cases of COVID-19.

### Sample Definition

For sample calculation, we considered the staff of nurses working on the frontline against COVID-19 at the two university hospitals, which are composed of 133 professionals, 90 from H1 and 43 from H2. A total of 101 nurses participated in this study, selected through non-probabilistic convenience sampling, in order to reach the largest number of participants. StatCalc from the EpiInfo program, version 7, was used, using a confidence level of 95%, which required a minimum sample of 99 participants, with a minimum of 50% plus one participant from each institution.

### Selection Criteria

To select participants, it was used as inclusion criteria to be a nurse directly involved in nursing care or management of units that provide care to patients with COVID-19. As exclusion criteria, nurses who were working remotely due to risk factors related to the pandemic and the absence of participants due to vacation, leave or benefit were considered.

### Data Collect

Data collection took place after approval by the Research Ethics Committee, from February to May 2022, online, through the free and free digital technology of Google Docs from Google Company Inc. and after authorization by the teaching and research management of the two selected university hospitals. To recruit participants, an invitation was sent via email. Email content included a description of the research proposal, its respective objectives, a link to access the instrument, instructions and deadline (four weeks) for completing it online. Weekly reminders were also sent by email recalling the importance and contribution to developing the research.

Then, the link with the Informed Consent Form (ICF) was forwarded, pointing out the objective and other ethical precepts as a guarantee of anonymity. The research instrument could only be completed after nurses expressed acceptance. Those who agreed to participate in the research signed the ICF, which was registered.

### Data Collection Instrument

For data collection, a questionnaire composed of two parts was used. The first part had semi-structured, mixed questions that made it possible to identify participant characteristics, such as age, sex, marital status, maximum title, length of professional experience, whether they had children or lived with family members in the risk group for COVID-19 and variables regarding their performance during COVID-19.

The second part consisted of the developed questionnaire^(22)^, Ethical issues Experienced by Nurses in Emergency Questionnaire, adapted and validated for the Brazilian context in accordance with international guidelines^(23)^.

The instrument seeks to investigate the ethical issues experienced by nurses in the context of health emergencies, based on a five-point Likert scale with response intervals ranging from one (“completely disagree”), two (“strongly disagree”), three (“neither disagree nor agree”), four (“strongly agree”) and five (“completely agree”), composed of 16 questions, and all results are summed, producing an overall score of ethical issues^(22)^.

The data obtained were subjected to factorial analysis, and the results were grouped into five groups of responses called constructs. The instrument’s level of reliability was verified through composite reliability, which presented an internal consistency value of 0.86. Construct coefficients presented values between 0.76 and 0.87, and Barlett’s sphericity test (BTS) identified a statistical significance of 0.000, proving the reliability of the generated constructs.

The pre-test of the validated version was applied to a sample of 32 graduate students, 10 master’s and 22 doctoral students, and 3 nurses working at two teaching hospitals in southern Rio Grande do Sul, recruited online. In this phase, no adjustments were necessary, as less than 10% of participants reported doubts about the items^(23)^. Participants reported a mean of 12–15 minutes to complete the survey.

### Data Analysis

Data analysis was performed by double typing in Microsoft Excel 2016 and, later, they were entered into the Statistical Package for the Social Sciences (SPSS) version 23. Descriptive statistical analysis was performed through frequency distribution and measurement of position. Data normality was verified using the Kolmogorov-Smirnov test and homoscedasticity using the Levene test^(24)^.

Therefore, the following parametric statistical tests were performed: Pearson’s correlation test (considering a very strong association variation between 0.91 and 1.00; strong association, from 0.71 to 0.90; moderate association, from 0.41 to 0.70; weak association, between 0.21 and 0.40; very weak association, from 0.01 to 0.20) for the association between the variables age and I worry about being infected with COVID-19 with the instrument factors. The regression analysis sought to assess which factors have the greatest effect on nurses’ ethical issues. P-value <0.05 was used as statistical significance for all analyzes^(24)^.

### Ethical Aspects

Ethical aspects were fully respected, in accordance with Resolution 466/12, the project being previously approved by the Research Ethics Committee, under Opinion 5.074.202/2021 and the collection by signing the ICF.

## RESULTS

The study included 101 nurses with a mean (M) age of 39.9 years (standard deviation (SD) = 7.8). The individuals were predominantly female (84.2%; 85), married (41.6%; 42) and with children (68.3%; 69).

Regarding professional activity, the mean time of professional activity was 14.5 years (SD = 7.75). Furthermore, most nurses (48.5%; 49) have specialization, followed by (29.7%; 30) master’s and (9.9%; 10) doctoral degrees. Regarding the COVID-19 infection, 55.4% (56) reported having been infected and 95% (96) were vaccinated with 3 doses of the vaccine against COVID-19. The sociodemographic profile and individuals’ work characteristics are detailed in Table 1.

**Table 1:** Characterization of nurses according to sociodemographic and work variables – Rio Grande, RS, Brazil, 2020 (N = 101).

Variables	Categories	N	%
**Age**	24 – 29 years	7	6.9
	30 – 39 years	51	50.5
	40 – 49 years	31	30.7
	50 – 59 years	9	8.9
	60 years or older	3	3
**Sex**	Female	85	84.2
	Male	16	15.8
**Marital status**	Single	31	30.7
	Married	42	41.6
	Stable union	30	19.8
	Divorced	8	7.9
**Have children**	Yes	69	68.3
	No	32	31.7
**Length of professional experience**	01 – 5 years	10	9.9
	06 – 10 years	20	19.8
	11 – 15 years	37	36.6
	16 – 20 years	10	9.9
	21 – 25 years	17	16.8
	26 years or older	7	6.9
**Acting hospital**	H1	79	78.2
	H2	22	21.8
**Maximum title**	Undergraduate degree	06	5.9
	Specialization	49	48.5
	Residence	06	5.9
	Master’s degree	30	29.7
	Doctoral degree	10	9.9
**Tested positive for COVID-19**	Yes	56	55.4
	No	45	44.6
**I received the COVID-19 vaccine**	1 dose	1	1
	2 doses	3	3
	3 doses	96	95
	Did not vaccinate	1	1
**I worry about being infected with COVID-19**	Yes	98	97
	No	3	3

Through descriptive analysis (Table 2), it was possible to identify the relationship between mean and standard deviation of constructs and instrument items. The mean number of ethical issues in patient care (F1) was 3.38 (SD = 1.02). The items with the highest mean were: question one: I am concerned about providing care to patients with COVID-19 due to the high degree of infectivity (3.83); question three: it is stressful and challenging for me to provide care to patients with COVID-19 (3.52); and question two: if I can care for patients with COVID-19 and patients with other pathologies, I will care for patients with other pathologies (2.73).

**Table 2:** Mean ( $\bar{\text{X}}$ ) and standard deviation (SD) of constructs and questions related to ethical issues in patient care during COVID-19 and associated factors. Rio Grande, RS, Brazil, 2022 (N = 101).

Factors	N	$\bar{\text{X}}$	SD
***F1 – ETHICAL ISSUES IN PATIENT CARE***	**101**	**3.38**	**1.023**
**Q01. I AM CONCERNED ABOUT PROVIDING CARE TO PATIENTS WITH COVID-19.**	101	3.83	1.217
**Q02. IF I HAVE TO CHOOSE BETWEEN COVID-19 PATIENTS AND OTHER TYPES OF PATIENTS, I WILL CARE FOR OTHER TYPES OF PATIENTS.**	101	2.73	1.288
**Q03. IT WILL BE STRESSFUL FOR ME TO CARE FOR PATIENTS WITH COVID-19.**	101	3.52	1.154
***F2 – PERCEIVED RISK OF INFECTION AND WILLINGNESS TO WORK***	**101**	**1.57**	**.650**
**Q04. IF NOT REQUESTED BY COVID-19 PATIENTS, I WILL NOT PROVIDE ADDITIONAL CARE MYSELF.**	101	1.86	1.059
**Q05. THERE IS A NEED TO REDUCE THE HOLISTIC CARE PROVIDED TO PATIENTS WITH COVID-19.**	101	1.49	.743
**Q07. IF I HAVE TO TAKE CARE OF COVID-19 PATIENTS EVERY DAY, I WILL QUIT MY JOB.**	101	1.56	.888
**Q08. I WOULD LIKE TO CHANGE PROFESSIONS BECAUSE OF COVID-19.**	101	1.29	.589
***F3 – PERCEPTION OF SOCIAL STIGMATIZATION***	**101**	**2.45**	**1.050**
**Q10. IF PEOPLE WERE AWARE OF THE FACT THAT I AM PROVIDING CARE TO PATIENTS WITH COVID-19, I MIGHT HAVE DISADVANTAGES.**	101	3.15	1.299
**Q11. I AM AFRAID OF BEING ISOLATED IF MY NEIGHBORS FIND OUT THAT I CARE FOR COVID-19 PATIENTS.**	101	2.10	1.136
**Q12. I AM CONCERNED THAT MY FAMILY WILL BE ISOLATED IF MY NEIGHBORS FIND OUT THAT I CARE FOR COVID-19 PATIENTS.**	101	2.12	1.160
***F4 – AGREEMENT WITH INFECTION CONTROL MEASURES***	**101**	**4.46**	**.500**
**Q13. WHEN I SEE A PATIENT WITH RESPIRATORY ILLNESSES ACCOMPANIED BY FEVER, I CHECK WHETHER PATIENTS HAVE COME FROM A HOSPITAL WITH SUSPECTED COVID-19 INFECTION, HAVE HAD CONTACT WITH A PATIENT WITH COVID-19, OR HAVE RECENTLY TRAVELED.**	101	4.10	1.118
**Q14. I WASH MY HANDS BEFORE AND AFTER HAVING CONTACT WITH A PATIENT WITH COVID-19 OR AN INFECTIOUS SUBSTANCE AND BEFORE AFTER PUTTING ON MY PERSONAL PROTECTIVE EQUIPMENT.**	101	4.85	.410
**Q17. WHEN COUGHING, I COVER MY MOUTH AND NOSE WITH A TISSUE, AND DISCARD IT IN AN APPROPRIATE PLACE.**	101	4.22	1.035
**Q18. I WASH MY HANDS IF I COVER MY MOUTH WITH MY HANDS WHEN I COUGH.**	101	4.66	.637
***F5 – PERCEPTION OF HOSPITAL MEASURES AGAINST COVID-19***	**101**	**3.26**	**.974**
**Q19. MY HOSPITAL IS EQUIPPED WITH SUFFICIENT FACILITIES TO PREVENT THE SPREAD OF COVID-19.**	101	3.14	1.158
**Q20. MY HOSPITAL FOLLOWS THE BEST INFECTION CONTROL GUIDELINES TO PREVENT THE SPREAD OF COVID-19.**	101	3.49	1.128
**Q21. MY HOSPITAL REGULARLY DISCUSSES HOW TO PREVENT COVID-19.**	101	3.15	1.152

$\bar{\text{X}}$ – mean; SD – standard deviation.

The construct “agreement with infection control measures against COVID-19” (M = 4.46; SD = .500) had the highest mean among the constructs. The item “I wash my hands before and after having contact with a patient with COVID-19 or an infectious substance and before after putting on my personal protective equipment” had the highest mean among the questions (4.85). It was evident that professionals realize the importance of following the recommendations to prevent the spread of the virus and cross-infection.

The second construct with the highest mean was “perception of hospital measures against COVID-19” (M = 3.26; SD = .974), identifying that nurses perceive that the work ­institution followed the norms and adaptations to face the emergency international public health. The item “my hospital follows the best infection control guidelines to prevent the spread of COVID-19” had the highest mean (3.49), followed by “my hospital regularly discusses how to prevent COVID-19” (3.15).

The construct “perception of social stigmatization” obtained a mean of 2.45 (SD = 1.05), with the question “if people were aware of the fact that I am providing care to patients with COVID-19, I might have disadvantages” (3.15).

The nurse realizes that he may be excluded or suffer some sort of prejudice if people know that he works in a hospital during COVID-19. The construct “perceived risk of infection and willingness to work” had a mean of 1.57 (SD = 1.113). The item “if not requested by COVID-19 patients, I will not provide additional care myself” (1.89) was the item with the highest mean, demonstrating that, no matter how stressful and fearful situations are manifested intensely, professionals feel a moral obligation to develop care.

Through Pearson’s correlation, a significant and moderate correlation was identified between the variable “I worry about being infected with COVID-19” with constructs F1 – Ethical issues in patient care (r = .527; p = .022) and F4 – Agreement with infection control measures (r = 596; p = .049), with a ­reference value for moderate association 0.41 – 0.70.

To determine the factors that influence nurses’ ethical issues during COVID-19, this study entered the main variables (ethical issues in patient care, perceived risk of infection and willingness to work, perception of social stigmatization, agreement with infection control measures, and perception of hospital measures against COVID-19) in a multiple linear regression analysis using the Enter method.

The analysis resulted in a statistically significant model (F (4.96) = 5.608; p < 0.001; R2 .661), showing that participants’ ethical issues were influenced by all constructs, being more affected by the perception of social stigmatization (β = .376, p = .003). Participants’ ethical issues were also high when there was no perception of infection control measures at work (β = .373, p = .000) (Table 3).

**Table 3:** Linear regression of the factors that impact nurses’ ethical issues during COVID-19. Rio Grande, RS, Brazil, 2022 (n = 101).

Variables	Beta (β)	T	p
**F2 – Perceived risk of infection and willingness to work**	.178	2.653	.009*
**F3 – Perception of social stigmatization**	.376	5.778	.003*
**F4 – Agreement with hospital infection control measures**	.172	2.760	.007*
**F5 – Perception of hospital measures**	.373	5.792	.000*

* Significance level p < 0.05.

Additionally, the test obtained an adjusted determination coefficient (R2) of .661, representing 66% of the explanation of the variation of factors related to nurses’ ethical issues during COVID-19. With regard to Durbin-Watson statistics, the value obtained was 1.771 in the error autocorrelation test for regression analysis, indicating that there was no autocorrelation. Furthermore, the absence of multicollinearity was identified, with a tolerance for the test of .847 – .982, greater than 0.1 and a variance inflation factor (VIF) of 1.018 – 1.180, lower than the reference level of 10. As for the absence of outliers, it presented residual statistics of –2.183 – 2.443, within the reference value of –3 and 3.

## DISCUSSION

The results of this study showed that the most common ­ethical issue experienced by nurses was related to concern, stress and tension in providing care to patients infected with COVID-19, demonstrating that nurses have a mindset to accept and declare their willingness to, if possible, avoid developing care for patients infected by COVID-19, giving preference to caring for non-infected patients. In addition to this, it was identified that the occurrence of ethical issues experienced by professionals was more influenced by perception of social stigma.

A study carried out in China with nurses and nursing ­students showed that frontline nurses exhibit anxiety, fear, ­sadness and anger, as they carry out their activities in the context of a pandemic^(11)^. Therefore, identifying nurses’ ethical issues during COVID-19 is of fundamental importance for professionals to feel more welcomed and better prepared to act and make decisions in future emergencies.

Until the appearance of the first patient, COVID-19 was a totally unknown disease worldwide. As expected, the number of infected rose rapidly and, globally, the nation was gripped by anxiety, leading to a socioeconomic depression^(25)^.

As countries, public health professionals and policy makers moved to address COVID-19, morally difficult ethical and social decisions and implications emerged. Among them, it is worth highlighting the professional and ethical duties of treating and caring for the serious risk to health and personal danger for care teams, especially nurses, who develop full-time care, exposed to a high viral load. Therefore, care has become extremely stressful and tense, resulting in ethical issues in nurses’ work^(26)^.

The factor with the highest mean between the constructs was agreement with infection control measures (M = 4.46). This finding is consistent with findings from studies conducted in China^(12)^, Israel^(26)^ and Egypt^(27)^, in which the infection prevention and control behaviors of healthcare workers working in hospitals have vastly improved, directly impacting the potential risk of COVID-19. Following infection control measures contributed to a greater general sense of competence of nurses in providing safe, humane and effective care, directly affecting their willingness to work and ethical decision-making in the face of the problems faced.

Faced with the emergence of a contagious disease, the importance of support from the government, hospitals and management to establish precautionary protocols against the infectious disease as well as to promote education through training for nurses is evident., to reinforce their infection prevention behavior^(15)^, resulting in the minimization of nurses’ ethical issues in caring for patients in emergency situations.

In this study, the second construct with the highest mean was F5, showing that professionals identify institutional commitment to providing resources or organizational processes to help them with their ethical concerns. These findings corroborate an Israeli study^(26)^, which aimed to assess how nurses respond to tension and ethical dilemmas during COVID-19, which evidenced a significant correlation in the existence of political guidelines for the treatment of patients with COVID-19 at interviewees’ workplace (p = 0.02), or having received specific training on the subject (p = 0.00) in that refers to recommendations for controlling the spread of the virus.

In this research, the perceived risk of infection and willingness to work presented a mean of 1.57 responses. According to research^(28)^, initially, the degree of perceived risk of nurses to contract COVID-19 was much higher than thorough the pandemic. This fact was probably due to conflicting messages given by the media as well as real-time updates about the disease, its progress, the availability of protective measures and their effectiveness. This result leads us to believe that, as professionals became more familiar with the disease, their emotional burden of risk perception decreased.

On the other hand, the variable “I worry about being infected with COVID-19” showed a statistically significant correlation with constructs F1 and F3, showing that the increase in perceived risk and concern with the care of patients infected with COVID-19 is related to the way in which health professionals adopt appropriate preventive measures more effectively against the disease. Corroborating with quantitative research with health professionals in Ethiopia, aiming to assess protective behaviors, risk perceptions and concern about COVID-19, it was identified that the higher the level of concern and perception of risk of infection, the more evident professionals’ motivation to adopt adequate preventive measures^(13)^.

Moreover, F1 and F3 had a greater influence on nurses’ ethical issues, demonstrating that professionals experience ethical issues in the face of fear of becoming infected and of suffering prejudice or that their family is harmed, if they know that they carry out their activities on the front line against COVID-19. From this perspective, a qualitative investigation, with the objective of determining the experiences and psychosocial problems among nurses in Turkey who work with COVID-19, reported that stigma was one of the ethical issues with a social effect identified by participants^(16)^.

Ethical issues related to stress, fear, uncertainty and stigma are supposedly common in health emergency situations and, therefore, require implementing interventions to promote mental health to face COVID-19 and other adverse health events^(19,29,30)^.

The results of this study also showed that F5 also had a significant influence on nurses’ ethical issues. This result corroborates research findings with health professionals who work on the front lines against COVID-19, showing that factors such as lack of material resources, low staffing, high risk of becoming infected and fear of transmitting to family members were listed as the main ethical issues related to fear and anxiety experienced by professionals^(30)^.

Although nurses experience ethical issues when they perceive the high risk of infection when providing care, it is not easy for them to abandon their moral obligation as professionals to promote care^(26)^. This is in line with this study, which presented F2 as the lowest mean construct. Therefore, it is fundamental to support and implement structural policies, protocols, permanent education and training with an emphasis on autonomy and recognition of professionals’ concerns so that nurses can develop patient care in safe and humanized environments.

The findings of this study contribute to clinical practice by providing useful information about the ethical issues faced by nurses during the pandemic and encouraging management to develop strategies that can minimize the occurrence of problems and provide support for coping with other public health emergencies in national and international importance.

The limitation of this study is the generalization of its results, because it was carried out with a specific sample of nurses from two university hospitals, selected by convenience sampling during COVID-19. Furthermore, the study analyzed only four variables that can influence nurses’ ethical issues, but there may be other variables that influence health care in other contexts. Thus, more research is needed to analyze influencing factors, in addition to the general characteristics and variables used in this study.

## CONCLUSION

In this study, it was possible to analyze that the most common ethical issue experienced by nurses was related to concern and stress in providing care to patients infected with COVID-19 and that the ethical issues experienced by nurses were more affected by social stigmatization perception. These results highlight the importance of encouraging professionals to take active precautions against infections in their care work, which will directly affect their willingness and confidence to provide humanized and qualified care.

Therefore, it is important to promote appropriate public, institutional and managerial awareness, reinforcing the need to provide health professionals with favorable work environments. Moreover, spaces should be offered so that nurses can reflect and discuss the ethical issues that permeate the nursing experience, not only in the context of a pandemic, but also in their care work, in order to encourage them to develop greater ethical skills to face conflicts and dilemmas, making them better prepared to carry out fair, prudent and empathetic decision-making in the face of the problems experienced.
